# Fosfomycin (Veterinary Medicinal Products): Summary

**DOI:** 10.14252/foodsafetyfscj.D-19-00020

**Published:** 2019-12-27

**Authors:** Food Safety Commission of Japan

## Abstract

The Food Safety Commission of Japan (FSCJ) conducted a risk assessment of fosfomycin (CAS No. 23155-02-4), using documents submitted for the ‟re-examination” of veterinary medicinal products. The data used for the assessment include ADME of fosfomycin Ca, residue of fosfomycin Ca and of fosfomycin Na, acute toxicity of fosfomycin Ca and fosfomycin Na. Data on subacute toxicity, reproductive and developmental toxicity, genotoxicity and microbiological effect are also included. The lowest-observed-adverse-effect level (LOAEL) in these toxicity studies was 175 mg (titer)/kg body weight (bw) per day based on adverse effects including diarrhea, autopsy findings such as erosion, hyperplasia, and exfoliation of glandular stomach mucosa, and histopathological findings such as erosion of the stomach and ileum mucosa observed as adverse effects in the 35-day subacute toxicity study in rats. Fosfomycin was not teratogenic in rats and rabbits. FSCJ considered that fosfomycin is not a genotoxic carcinogen. The toxicological ADI of fosfomycin was established to be 0.175 mg/kg bw per day by applying a safety factor of 1000 based on the LOAEL of 175 mg (titer)/kg bw per day. On the other hand, the microbiological ADI was calculated to be 0.019 mg/kg bw per day, which is lower than the toxicological ADI. Thus, FSCJ established the ADI for fosfomycin as 0.019 mg/kg bw per day.

## Conclusion in Brief

The Food Safety Commission of Japan (FSCJ) conducted a risk assessment of fosfomycin (CAS No. 23155-02-4), using documents submitted for the “re-examination” ^1^^)^ of veterinary medicinal products.

Fosfomycin is a distinct type of antimicrobial used as a veterinary medicinal product to treat *Escherichia coli* diarrhea and salmonellosis in cattle, and pseudotuberculosis of Perciformes in Japan. Fosfomycin calcium (fosfomycin Ca) is used as an additive in feed or drinking water, and fosfomycin sodium (fosfomycin Na) is used as an injection.

The data used for the assessment include ADME of fosfomycin Ca (rats, rabbits, dogs, cattle and yellowtails), residue of fosfomycin Ca (cattle and yellowtails) and of fosfomycin Na (cattle), acute toxicity of fosfomycin Ca and fosfomycin Na (mice and rats). Data on subacute toxicity (rats, rabbits and dogs), reproductive and developmental toxicity (rats and rabbits), genotoxicity and microbiological effect are also included.

## 1. Toxicological Effects

### a. Subacute Toxicity

FSCJ evaluated the subacute toxicity based on the submitted data of 35- or 182-day toxicity studies in rats, a 35-day toxicity study in rabbits, and a 182-day toxicity study in dogs. The lowest-observed-adverse-effect level (LOAEL) in these toxicity studies was 175 mg (titer)/kg body weight (bw) per day based on adverse effects including diarrhea, autopsy findings such as erosion, hyperplasia, and exfoliation of glandular stomach mucosa, and histopathological findings such as erosion of the stomach and ileum mucosa observed as adverse effects in the 35-day subacute toxicity study in rats. No-observed-adverse-effect level (NOAEL) was not obtained for subacute toxicity.

### b. Reproductive and Developmental Toxicity

There is no study available for the two-generation reproductive toxicity. In gavage administration study during the period of organogenesis in rats treated during gestation (day 7 to 17) dams had loose stools, and fetuses showed increased early resorptions and delayed ossification after administration of 1400 mg /kg bw per day. One third of dams were allowed for parturition. No treatment-related effect was observed on offspring. The NOAELs for both dams and embryo/fetuses were considered 700 mg (titer) /kg bw per day. In a study of rabbits treated during gestation (day 6 to 18), none of adverse effects were observed in the dams and fetuses at all the doses examined. Therefore, the NOAEL for both maternal and embryo/fetus toxicity was considered to be 420 mg/kg bw per day, the highest dose tested.

Fosfomycin was not teratogenic in rats and rabbits. The lowest value of NOAEL was 420 mg/kg bw per day obtained in dams and fetuses of rabbits.

### c. Genotoxicity/carcinogenicity

The results of genotoxicity studies, including reverse mutation, DNA damage and mutation tests were negative. The results of both dominant lethal and micronucleus tests in rodents were also negative. Hence, FSCJ judged that fosfomycin had no genotoxicity relevant to human health.

In spite of the lack of chronic toxicity and carcinogenicity studies, toxicological effects, suggesting cytotoxicity and increased cell proliferation were not observed in 182-day administration studies in rats and dogs. FSCJ thus considered that fosfomycin is not a genotoxic carcinogen.

### d. Toxicological ADI

FSCJ judged to be able to specify the acceptable daily intake (ADI), since fosfomycin is not a genotoxic carcinogen. Among NOAELs and LOAELs from the toxicity studies evaluated, the lowest value was the LOAEL of 175 mg/kg bw per day in a 35-day subacute toxicity study in rats. FSCJ considered it appropriate to specify the ADI based on this LOAEL.

Although the chronic toxicity study was not conducted, observed toxic effects did not differ obviously between 35- and 182-day subacute toxicity studies thus prolonging of the administration period did not enhance the toxic effect.

Teratogenicity was not observed in the administration studies in rats and rabbits, and no effects were observed on the reproductive potential of the dams, although two-generation reproduction study was not conducted.

Therefore, FSCJ concluded that an additional 10 to a safety factor 100 was necessary to specify ADI based on the following reasons:

1) The use of the LOAEL in the 35-day subacute toxicity study in rats

2) Six-day, instead of seven-day, per week in the daily administration in the fosfomycin study

3) No studies for assessing chronic toxicity and carcinogenicity

As the results, the toxicological ADI of fosfomycin was established to be 0.175 mg/kg bw per day by applying a safety factor of 1000 (i.e. species difference of 10, individual difference of 10, and additional factor of 10) based on the LOAEL of 175 mg (titer)/kg bw per day in the 35-day subacute toxicity test in rats.

## 2. Microbiological ADI

FSCJ considered to use detailed data for microbiological ADI, which are obtained from “Microbiological effects of veterinary antibacterial agents” (FSCJ Contracted Survey Program 2006, #cho20070330013). The data were sufficient for estimation of microbiological ADI based on the VICH (the International Cooperation on Harmonization of Technical Requirements for Registration of Veterinary Medicinal Products) guideline 36. To calculate a microbiological ADI, FSCJ applied 0.004397 mg/ml of calculated minimum inhibitory concentration (MICcalc), 84% indicating fraction of exposed bacteria based on the urine recovery of 16.4% until 24 h after 500 mg (titer) administration in humans, 220 g for mass colon content, and 60 kg as body weight of a person to the VICH formula as follows:

ADI (mg/kg bw/day) = 0.004397*1× 220*2(1−0.16)*3× 60*4 = 0.01919 mg/kg bw/day

*1: The MICcalc is derived from the lower 90% confidence limit for the mean MIC_50_ of the relevant genera for which the drug is active, as described in the VICH guidelines.

*2: Mass of colon content (g)

*3: Ratio of biologically available oral dose that is estimated from the urine excretion rate of 16.4% of total dose in an oral administration test in humans.

*4: Body weight of person (kg).

## 3. ADI Establishment

The ADI for fosfomycin can be established since fosfomycin is not a genotoxic carcinogen. FSCJ compared the toxicological ADI (see the section 1.d.) with the microbiological one.

FSCJ established the toxicological ADI to be 0.175 mg/kg bw per day, as was mentioned above. The microbiological ADI was established to be 0.019 mg/kg bw per day, which is lower than the toxicological ADI. Thus, FSCJ proposed the ADI as 0.019 mg/kg bw per day.

## 4. Conclusion

On the basis of the above assessment, FSCJ established the following ADI for fosfomycin:

Fosfomycin: 0.019 mg/kg bw/day

(table 1)

**Table 1:** Levels relevant to toxicological evaluation of Fosfomycyn

Species	Study	Dose (mg/kg bw/day)	NOAEL (mg/kg bw/day)
Rat	35-day subacute Toxicity study	0, 175, 350, 700, 1400, 2800	175 (LOAEL) Diarrhea, erosion, hyperplasia, and exfoliation of glandular stomach mucosa Erosion of the stomach and ileum mucosa (M)
	182-day subacute Toxicity study	M: 0, 87.5, 175, 350, 700, 1400	700 Vacuoles in hepatocytes
	Reproductive and developmental toxicity (Administration study during the period of organogenesis)	0, 140, 700, 1400	Dams and fetuses: 700 Loose stools (dams) Increased early resorptions and delayed ossification (F_1_) (No teratogenicity)
Rabbit	35-day subacute Toxicity study	M: 0, 200, 400	200 Increase in T.chol
	Reproductive and developmental toxicity (Administration study during the period of organogenesis)	0, 80, 140, 420	Dams and fetuses: 420 (No teratogenicity)
Dog	182-day subacute Toxicity study	F: 0, 280, 560	280 (LOAEL) Diarrhea, Suppression of body weight gain and smaller amount of food consumption Increase in serum Ca and InP Hypertrophy of the liver, kidney congestion and caecaum distention Swollen renal tubular epithelial cell
Toxicological ADI (mg/kg bw/day)			0.175 LOAEL: 175 SF: 1000
The critical study for setting Toxicological ADI			35-day subacute toxicity study in rats
Microbiological ADI (mg/kg bw/day)			0.01919
The critical study for setting Microbiological ADI			The value was specified in accordance with the VICH guideline. FSCJ had obtained adequate evidence to apply the MICcalc of fosfomycin against human clinically isolated strains in a comprehensive survey of the emerging problems for ensuring food safety in FY 2006.
ADI (mg/kg bw/day)			0.019

M, Male; F_1_, F_1_ generation; ADI, Acceptable daily intake; NOAEL, No-observed-adverse-effect level; SF, Safety factor
